# Beyond milestones: reconstructing the foundations of JBRA Assisted Reproduction

**DOI:** 10.5935/1518-0557.20260043

**Published:** 2026

**Authors:** Edson Borges Jr.

**Affiliations:** 1 Fertility Medical Group/FERTGROUP Medicina Reprodutiva, Sao Paulo, Brazil; 2 Instituto Sapientiae - Centro de Estudos e Pesquisa em Reprodução Assistida

The recent editorial celebrating three decades of JBRA Assisted Reproduction (JBRA) appropriately highlights the journal’s role in advancing reproductive medicine in Latin America and its progressive internationalization (Taitson *et al*., 2026). As the journal continues to consolidate its global presence, it is both timely and necessary to revisit the foundations that enabled this trajectory.

The origins of JBRA are closely linked to the vision of its founding editor, **Dr. José Gonçalves Franco Júnior**, whose early leadership helped define the journal’s mission and direction. In its inaugural editorial - “Um jornal, uma filosofia, uma esperança…” (“A Journal, a Philosophy, a Hope…”)- he articulated a clear purpose: to discuss, disseminate, and seek solutions to the challenges of assisted reproduction, within a scientific environment grounded in clear rules and minimal political interference (Franco Jr, 1997). This statement established not only the identity of a new journal, but also a guiding framework that would shape its long-term development.

From its inception, JBRA was conceived as a collective scientific endeavor. The editorial structure of its first issue reflected a broad and engaged community, with the participation of associate editors including Maria do Carmo Borges, Edson Borges Jr, Ricardo Baruffi, Selmo Geber (in memoriam), and Weydson Barros Leal (in memoriam), João Batista Oliveira, among many others, alongside an extensive editorial board composed of leading figures in Brazilian reproductive medicine. This collaborative foundation ensured diversity of perspectives and contributed to the establishment of a consistent and credible scientific platform at a formative stage.

Over the following decades, the development of JBRA closely paralleled the maturation of assisted reproduction in Brazil and across Latin America. The consolidation of its editorial structure, the expansion of its scientific scope, and the progressive engagement of both regional and international communities contributed to building a robust publication platform.

A defining inflection point in this trajectory was the transition toward digitalization and international accessibility. This transformation was formally articulated in later editorial reflections, which emphasized the need to increase visibility, expand international reach, and transition to an English-language, open-access model as a prerequisite for global integration (Franco Jr, 2014). The strategic pursuit of indexing in PubMed emerged within this context as a key objective for positioning Latin American research within the global biomedical literature.

The subsequent inclusion of JBRA in major indexing systems including PubMed, Web of Science, and Scopus, represents a defining milestone in its history. These achievements enhanced the journal’s visibility and citation pathways, while reflecting a long-term editorial strategy developed over time. This advancement was made possible through the sustained commitment and leadership of **Dr. José Gonçalves Franco Júnior**, whose efforts together with those of his editorial team, were instrumental in achieving this level of international recognition. As documented in institutional records and historical compilations of the Brazilian Association of Assisted Reproduction (SBRA), this evolution required continuous adaptation to rigorous quality standards and alignment with international publishing practices

Equally, this trajectory reflects the enduring contributions of editors, associate editors, members of the editorial board and, fundamentally, the authors who entrusted JBRA with the dissemination of their scientific work. Their collective commitment has been essential in building, consolidating, and projecting the journal within the international scientific community.

These contributions remain embedded in the journal’s identity and continue to support its ongoing development.
